# Motion Mode Recognition and Step Detection Algorithms for Mobile Phone Users

**DOI:** 10.3390/s130201539

**Published:** 2013-01-24

**Authors:** Melania Susi, Valérie Renaudin, Gérard Lachapelle

**Affiliations:** PLAN Group, Schulich School of Engineering, The University of Calgary, 2500 University Drive NW, Calgary, AB T2N 1N4, Canada; E-Mails: melania.susi@ieee.org (M.S.); valerie.renaudin@ifsttar.fr (V.R.)

**Keywords:** pedestrian navigation, handheld MEMS, step detection, mobile phone, gait analysis

## Abstract

Microelectromechanical Systems (MEMS) technology is playing a key role in the design of the new generation of smartphones. Thanks to their reduced size, reduced power consumption, MEMS sensors can be embedded in above mobile devices for increasing their functionalities. However, MEMS cannot allow accurate autonomous location without external updates, e.g., from GPS signals, since their signals are degraded by various errors. When these sensors are fixed on the user's foot, the stance phases of the foot can easily be determined and periodic Zero velocity UPdaTes (ZUPTs) are performed to bound the position error. When the sensor is in the hand, the situation becomes much more complex. First of all, the hand motion can be decoupled from the general motion of the user. Second, the characteristics of the inertial signals can differ depending on the carrying modes. Therefore, algorithms for characterizing the gait cycle of a pedestrian using a handheld device have been developed. A classifier able to detect motion modes typical for mobile phone users has been designed and implemented. According to the detected motion mode, adaptive step detection algorithms are applied. Success of the step detection process is found to be higher than 97% in all motion modes.

## 1. Introduction

A consequence of the booming market of smartphones and other types of Personal Digital Assistant (PDAs) is that the possibility of using mobile devices for locating a person is becoming more and more attractive for many applications. Among them are health care services, commercial applications and safety services. The trend is evolving from applications dedicated to professional market to the consumer market. This explains the increasing interest for research in handheld based positioning for Location Based Services (LBS) as it could broaden the offer of services based on user's locations. For example, mobile phone users can automatically receive information about places of interest, such as restaurants or shops according to their actual locations or they can be constantly updated about the location of their friends' network. Knowing a subject's location can also help to quickly provide assistance services in emergency situations or help in the assistance of elderly or impaired people. Subsequently, it is necessary to guarantee accuracy, continuity and availability of provided services in different environments. It is known that Global Navigation Satellite Systems (GNSS) are the primary source for tracking user's position. In indoor environments, in urban canyons and in other challenging surroundings the availability of satellite signals cannot be guaranteed and GNSS based services can be highly degraded or totally denied. In these cases, micro-electromechanical systems (MEMS), such as accelerometers and gyroscopes, can aid the geolocation process. The main advantage of using these sensors is that they are already embedded in most emerging mobile devices.

However, the main drawback of low grade MEMS sensors is that, since their signal is affected by various noises and drifts [1], they cannot constitute a self-contained system. In order to bound the above error sources, frequent GNSS updates can be used. When GNSS aiding is not available other approaches have to be investigated in order to geolocate users. A widespread approach consists in applying Pedestrian Dead Reckoning (PDR) using embedded inertial sensors. Contrary to strap-down navigation, PDR offers an interesting strategy because it exploits the kinematic of the human gait [2] instead of doubly integrating the inertial data. Given a starting known position, PDR algorithm propagates the user's position by estimating the heading and the user's travelled distance or the user's speed. Traditionally, PDR algorithms compute the travelled distance by detecting the user's steps and determining their length. This technique has been used for navigation application in many research works, as detailed in Section 2.

## 2. Background and Related Works

The PDR approach is very effective when the sensor is rigidly mounted on the user's body, especially when it is located on the user's foot. In this case, the stance phases of the foot (when the foot is flat on the ground) can be identified and associated with step events. Periodic zero velocity updates (ZUPTs) and/or zero angular rate updates (ZARUs) are then performed to bound the position error accumulation [3–5]. The same techniques can be adopted when the sensor is mounted on the user's leg or ankle. In fact, as in the above cases, quasi-static periods, even if shorter than the ones identified for the foot case, are still present [6]. The case of inertial sensors fixed on the user's torso or belt is also explored [7–10]. In the above situations, the inertial force experienced by the sensor is directly linked to the motion of the human body's Centre Of Mass (COM) and, subsequently, to the global user's motion. However, body fixed locations are not realistic for consumer grade applications since generic users usually carry their mobile devices in their hands, lower pockets or handbags. The case of unmounted sensors for inertial pedestrian navigation is only marginally explored. Some research deals with the case of the sensors in the user's pocket [11,12]. When the subject is walking with the sensor in the pocket, the IMU signal is generally undistorted and reflects the global user's motion. Subsequently, in this case, approaches similar to the body fixed case can be adopted. Indeed, a handheld mobile device can be subjected to various types of motions and the pattern of the sensor signal is less predictable than in the above mentioned cases. Gusenbauer *et al.* [13] analyse the situation where the user carries the mobile device in his/her hand looking at the screen to consult navigation instructions. The mentioned work does not consider different carrying modes, which is a limitation for realistic applications. Another promising approach for navigation purposes using handheld devices is based on the integration of inertial sensor signals with a camera, nowadays embedded in all smart phones [10,14,15]. This approach is based on specific assumptions regarding the light conditions and the camera orientation determined by the device orientation.

For devices carried without any assumption about their orientation or position the continuous identification of the device carrying mode and the user motion can help to constrain and properly tune the navigation algorithms according to the specific situation and, consequently, to the characteristics of the sensor signal pattern. For body fixed sensors, it has been shown that knowledge about the user motion can be exploited to aid PDR systems and refine the user's location estimate, e.g., [9,16]. The identification of human motion and gestures by using inertial sensors is an active and proliferating research field finding many applications included medical diagnosis, sport rehabilitation [17], elderly assistance [18], emergency services [19], monitoring systems [20], offender tracking [21], navigation [9,22] *etc.* Each application has specific requirements determining the number and type of sensors to be used, the sensor position and carrying mode, and the motions/activities to be recognized. For instance, the use of multiple sensors is a common approach for medical applications and rehabilitation purposes since more sources of information increase the accuracy of the classification process. The use of multiple body fixed sensors for human activity recognition has been deeply investigated in the literature. An extensive comparison of classification techniques using inertial body worn sensors can be found in [23–25]. In the recent years, human activity recognition by exploiting the capabilities of modern handheld smart phones or devices is also becoming an active field of research. However, the identification of human activities recognition by using mobile phones introduces many peculiar issues [26,27]. One of the main requirements is that the classification should be robust, regardless of the device carrying mode and its orientation. Some research work focuses on the identification of human activities using portable devices in order to localize users in the outdoors relying on the combination of GPS and other technologies or external source information, e.g., Geographic Information System (GIS) and inertial sensors data [28,29]. However these algorithms, which rely on GPS measurements, are designed for outdoor applications and, consequently, are not suitable for navigation purposes in satellite signal denied environments. A very few recent papers propose navigation algorithms for handheld devices based only on inertial sensors data and taking in account different sensor carrying modes including the swinging hand case. The latter is the most critical situation. Indeed, when an inertial sensor is held in a swinging hand, the hand motion can hide the global pedestrian motion making the step identification difficult. For this reason, the techniques used for “body fixed” sensors cannot be applied to the swinging case and a dedicate analysis is required. Recently Pei *et al.* [30] proposed a Least Square-Support Vector Machines for identifying eight motion states, considering different carrying modes including the swinging situation. Based on accelerations and magnetic field data recorded with a handheld unit, this classifier performs with successful recognition rates varying between 80.45% and 95.53% depending on the considered features. Then, human activity recognition is combined with wireless positioning for indoor navigation.

Kamisaka *et al.* [31] propose the design and implementation of a PDR system for mobile phone applications. This work deals with different carrying modes including the swinging case. The latter is distinguished from other sensor carrying modes by analysing the output of a magnetometer. Specifically, the magnetometer measurements are used to evaluate the variation in the angular difference detected by the sensor. This approach is motivated by the observation that arm swinging produces a sinusoidal magnetometer output. Once the specific carrying mode is identified, user steps are detected by exploiting the magnetometer outputs in the swinging case and the accelerometer outputs in all the other analysed carrying modes. However, magnetic fields experience rapid variations in indoor spaces due to surrounding ferromagnetic compounds, which may perturb the sinusoidal pattern analysis.

## 3. Motivation and Paper Outline

In this paper, different kinds of motions commonly experienced by handheld inertial sensors are considered and identified using a dedicated classifier. In particular, the case of an IMU carried in the user's swinging hand is distinguished from the situation when the user is interacting with the mobile device, for example when the walking subject is phoning or texting a message on his/her mobile device. In order to identify the swinging case, the proposed approach uses angular rates instead of magnetometer measurements exploited in [30,31]. Indeed, this appears to be a more robust source of information. A further element of novelty is the analysis of “irregular motions”. The irregular motion class includes all motions inducing an inertial force on the sensor that does not reflect a real change of user's displacement. The identification of this type of motion, which is frequently neglected in PDR algorithms, is fundamental for navigation purposes in order to discard parasite motions in the navigation process. This aspect is also pointed out in [9] where a waist mounted IMU is exploited to detect different types of motions including irregular motions, such as “bending over”, which are not linked to a change of the user's geographical position. Such irregular motions are even more likely to occur when the sensor is freely carried in a hand, which is the case considered here, but are also more difficult to identify due to the possible decoupling between hand and body motions. Knowledge of the motion mode is then exploited to develop step detection algorithms that are adapted to the mode experienced by the user. In fact, as shown in [32], the algorithms proposed here can be exploited in conjunction with a step length model to effectively compute the user's linear travelled distance by using handheld devices freely carried by the user. The challenging task of robustly detecting steps with handheld inertial sensors is essential for free-inertial PDR and is the motivation of this work.

The remainder of the paper is organized as follows: in Section 4, the signal model is introduced and in Section 5, the selected motion modes are illustrated. The features and the decision tree algorithm used for classifying the pedestrian and hand motion modes are described in Section 6. The step detection algorithm is described in Section 7. Section 8 details the data collections and assesses the proposed algorithms. Finally, Section 9 draws conclusions.

## 4. Signal and System Model

The motion mode recognition and the user's step recognition are performed using signals recorded with a six-degree-of freedom handheld Inertial Measurement Unit (IMU). The device is constituted by a tri-axis accelerometer and tri-axis gyroscope that respectively measure the acceleration and angular velocity of the rigid body with respect to the navigation frame. The output of the IMU **s** [n] ∈ ℝ^6^ can be modelled as the sum of the response to the experienced inertial force and a noise term [5]. It is composed by the accelerometer output **s***^a^* [n] ∈ ℝ^3^ and the gyroscope output and **s***^ω^* [n] ∈ ℝ^3^. It can be expressed as:

(1) $$s\left[\text{n}\right]=\left[\begin{array}{c} s^{\text{a}}\left[\text{n}\right]\\ s^{\omega }\left[\text{n}\right] \end{array}\right]=\left[\begin{array}{c} \text{a}\left[\text{n}\right]\\ \omega \left[\text{n}\right] \end{array}\right]+\left[\begin{array}{c} \eta^{\text{a}}\left[\text{n}\right]\\ \eta^{\omega }\left[\text{n}\right] \end{array}\right]$$

where:

- *n* ∈ ℕ represents the temporal index of the signal with sampling frequency f_s_ = 1/T_s_ equals to 100Hz,
- **a** [n] is the raw acceleration vector at the output of the tri-axis accelerometer,
- **η^a^** [n] is the noise vector associated to the accelerometer output,
- **ω** [n] is the raw angular rate vector at the output of the tri-axis gyroscope and
- **η***^ω^* [n] is the noise vector associated to the gyroscope output.

Due to their low grade nature, the sensors embedded in most common mobile devices are strongly affected by errors degrading their accuracy [1]. To cope with such errors, specific signal pre-processing is required as described in Section 6.1. In particular, the algorithms presented in this paper are extracting information from moving windows whose mean is removed in order to mitigate the slowly varying sensor errors, *i.e.*, the sensor bias drift.

## 5. Motion Mode Definition

As analysed in [27,33], portable handheld devices experience a large variety of motions producing different patterns in the recorded signals. The success rate of signal processing techniques for analysing pedestrian gait is therefore related to the design of adaptive analyses. Subsequently, knowing not only the user's global motion mode, but also his/her hand's motion is fundamental. Moreover it is important to identify all hand motions that do not reflect a real change in the user's location and should be only considered as perturbations for tracking the global displacement. Otherwise in these cases, the user position would be incorrectly propagated in any navigation filter. Such event occurs, for example, when the user is standing and looking for his/her phone in the bag. In these situations the subject is not changing his geographical position although a significantly inertial force is experienced by the handheld IMU.

In this paper, the following four different classes have been identified in order to better characterize the sensor carrying modes and motions typical for mobile phone users.

**Class 1**: this class includes all situations when user is static. The user is considered as static when his/her location does not change during the analysis temporal window. More specifically, it also includes the situation when the user is slightly moving but not significantly enough to be considered as a global locomotion. This is the case when the user is stepping on spot while answering a phone call. All these types of activities have to be recognized as static activities, so that the user position is not wrongly affected.
**Class 2**: this class refers to all cases when the device is quasi stable and the inertial force experienced by the sensor is mainly produced by the user global motion. It includes the following cases
  − Hand texting: in this case the mobile handheld device is almost stationary. For example, the user is walking, texting or reading a message on the phone. The situation when the subject is walking watching his/her mobile phone's screen to follow navigation instructions is included as well.
  − Hand phoning: the user walks while making or receiving a call.
  − Bag carrying: the user is walking with the mobile device in a bag held in one hand.
**Class 3**: it refers to the hand swinging case. The user is walking while holding the mobile device in his/her swinging hand.
**Class 4**: it includes the irregular motions. They are all motions that the user performs while he is standing and that do not contribute to the global locomotion. For example, the subject is searching the phone in a bag without walking.

## 6. Motion Mode Classification

The goal of a classification system is to automatically assign a given input pattern to known classes of objects, according to specific decision rules. In general, any classification process is performed according to the three following phases [34].

- Data pre-processing: the main purpose of this phase is to remove the noise from raw data enabling next phases.
- Features extraction and evaluation: this phase aims at extracting and evaluating meaningful parameters able to univocally characterize each class, therefore enabling the classification process.
- Decision Making: this phase refers to the last stage that is the association of the input pattern to a specific class.

**Figure 1.**
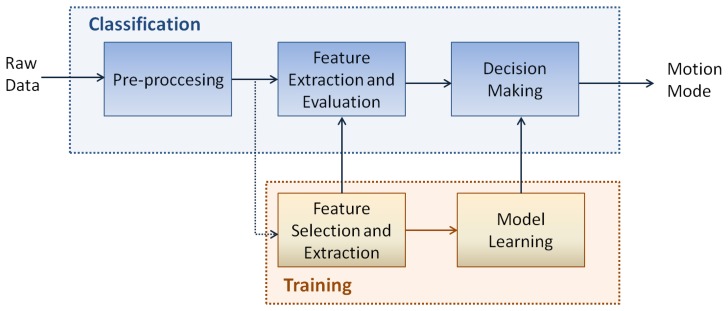
Scheme of a classifier for motion mode recognition.

As shown in Figure 1, where the general block scheme of a classifier for motion mode recognition is reported, the raw data are first pre-processed and then the extraction and evaluation of features is performed. The optimum features for characterizing the input pattern are found during the training phase. The latter has also the aim to learn the classifier according to a specific model. Finally, the class selection is performed. Following sections detail the above three phases that have been implemented for identifying the user's motion mode.

### 6.1. Pre-Processing

Since most of the energy captured by the accelerations and angular rates associated to human movements is below 15 Hz [35], the components in Equation (1) are first low-pass filtered using a 10th order Butterworth filter with a 15 Hz cut-off frequency. This filtering removes the high-frequency components of the noise. This phase is necessary for extracting useful information from the low-cost sensor signals enabling the human motion recognition process. It is worth pointing out that high-frequency components could be exploited to identify other types of contexts [36,37], such as driving a car, which are not considered in this paper.

Moreover processing is performed on the norm of the inertial signals. Indeed when the sensor is not body fixed, the orientation of the sensor cannot easily be determined, which affects the analysis of inertial signals along a specific axis. In addition, due to fast motions that are generally performed by hand, the orientation of the sensor can suddenly change and distorts a component-wise processing. To remove any dependence upon the sensor's orientation, the norm of the IMU signal is considered. The following notation is used to denote the filtered acceleration and angular rate norm:

(2) $${\tilde{s}}_{\text{rms}}\left[n\right]=\left[\begin{array}{c} {\tilde{s}}_{\text{rms}}^{a}\left[n\right]\\ {\tilde{s}}_{\text{rms}}^{\omega }\left[n\right] \end{array}\right]$$

where:

- ${\tilde{s}}_{\text{rms}}^{a}\left[n\right]=\sqrt{{\tilde{a}}_{x}^{2}\left[n\right]+{\tilde{a}}_{y}^{2}\left[n\right]+{\tilde{a}}_{z}^{2}\left[n\right]}$
- ${\tilde{s}}_{\text{rms}}^{\omega }\left[n\right]=\sqrt{{\tilde{\omega }}_{x}^{2}\left[n\right]+{\tilde{\omega }}_{y}^{2}\left[n\right]+{\tilde{\omega }}_{z}^{2}\left[n\right]}$

It has to be noted that the presence of a non-zero DC component can hide important information and reduce the effectiveness of frequency domain estimation techniques, which will be used later. Thus, the DC component is removed as follows:

(3) $${\tilde{s}}_{\text{rms}0}\left[n\right]={\tilde{s}}_{\text{rms}}\left[n\right]-\frac{1}{N}\sum_{l=0}^{N-1}{\tilde{s}}_{\text{rms}}\left[n-l\right]$$

The second term of Equation (3) is the signal mean computed using a moving average filter and *N* is the length of the moving average. The criterion used to select the analysis window length is explained in Section 6.2.

### 6.2. Feature Extraction

To perform any classification process a set of features has to be extracted. They are attributes able to characterize without ambiguity each motion mode. The features selection plays a key role in the entire classification process and strongly affects the final performance of the designed classifier. In particular, to reduce the probability of miss-classification, features have to be chosen in order to [34]:

- minimize the distance among different features belonging to the same class
- maximize the distance among different features belonging to different classes.

In this work, feature extraction is performed by dividing the norm of pre-processed inertial data in sliding windows of N samples with 50% overlap. The effectiveness of this choice for motion mode recognition has already been shown in previous work (e.g., [27,32]). The window's size has to be selected in order to be big enough for including at least a cycle gait but small enough to allow identifying sudden motion mode transitions. In this work N was chosen to be 256 samples that correspond to 2.56 seconds using a 100 Hz sampling frequency.

In addition, this size allows the fast computation of the Fast Fourier Transform (FFT) [38] used for the frequency analysis of the examined signals. The following features have been identified for the classification process:

- the gyroscope energy *E_s̃^ω^_*,
- the accelerometer energy *E_s̃^a^_*,
- the gyroscope variance σ*_s̃^ω^_*^2^,
- the accelerometer variance *σ_s̃^a^_*^2^, and
- the dominant frequencies of the gyroscope and accelerometer, respectively f*_s̃^ω^_* and f*_s̃^a^_*

They are described in the next sections.

#### 6.2.1. Signal Energy

**Figure 2.**
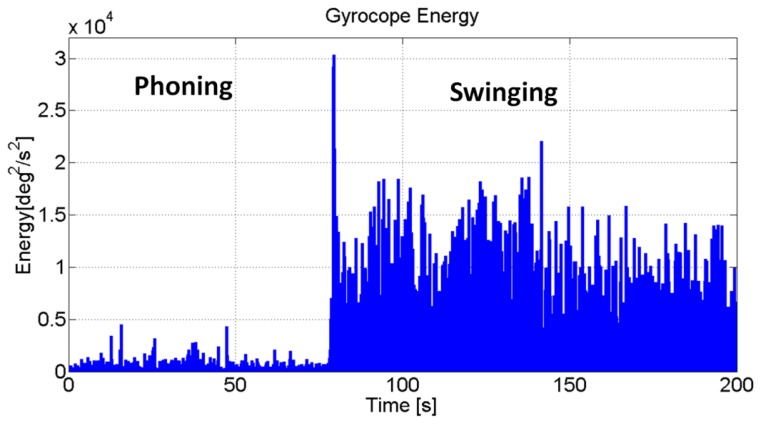
Energy of the gyroscope signal (norm) for a walking user with the IMU in the swinging and phoning hand.

Energy features allow distinguishing low and high intensity activities. The energy is computed here by squaring the norm of the accelerometer and gyroscope data and summing and normalizing them over a moving window as:

(4) $$E_{{\tilde{s}}^{a}}=\frac{1}{N}\sum_{n=0}^{N-1}{\tilde{s}}_{\text{rms}0}^{a}\left[n\right]$$

(5) $$E_{{\tilde{s}}^{\omega }}=\frac{1}{N}\sum_{n=0}^{N-1}{\tilde{s}}_{\text{rms}0}^{\omega }\left[n\right]$$

where *N* is the length of the analysis window and ${\tilde{s}}_{\text{rms}0}^{a}$ and ${\tilde{s}}_{\text{rms}0}^{\omega }$ are the norms, of the filtered accelerometer and gyroscope data. This feature mainly contributes to the identification of static and dynamic states. Figure 2 shows the results of an actual user walking along a straight line with the IMU alternatively in the phoning and swinging hand. The swinging mode experiences much higher amplitudes of the angular rate and acceleration energies as compared to the other states of interest, namely texting, phoning, bag carrying. It was found that in the latter cases, the motion sensed by the IMU is primarily due to the lower part of the body. For this reason, in the above situations, the sensor signals present patterns that are similar to the one experienced with body fixed. It was also found that texting, phoning and bag carrying present the same features and can be grouped in a unique state.

#### 6.2.2. Signal Variance

In order to improve the robustness of the classification process other features are considered. They are principally the variances of the gyroscope and accelerometer signals. The variance of a signal is a statistical measurement defined as the average of the squared differences from the mean. For the accelerometer and the gyroscope signal, they are expressed as:

(6) $$\sigma_{{\tilde{s}}^{a}}^{2}=\frac{1}{N}\sum_{n=0}^{N-1}{\left({\tilde{s}}_{\text{rms}0}^{a}\left[n\right]-\frac{1}{N}\sum_{n=0}^{N-1}{\tilde{s}}_{\text{rms}0}^{a}\left[n\right]\right)}^{2}$$

(7) $$\sigma_{{\tilde{s}}^{\omega }}^{2}=\frac{1}{N}\sum_{n=0}^{N-1}{\left({\tilde{s}}_{\text{rms}0}^{\omega }\left[n\right]-\frac{1}{N}\sum_{n=0}^{N-1}{\tilde{s}}_{\text{rms}0}^{\omega }\left[n\right]\right)}^{2}$$

**Figure 3.**
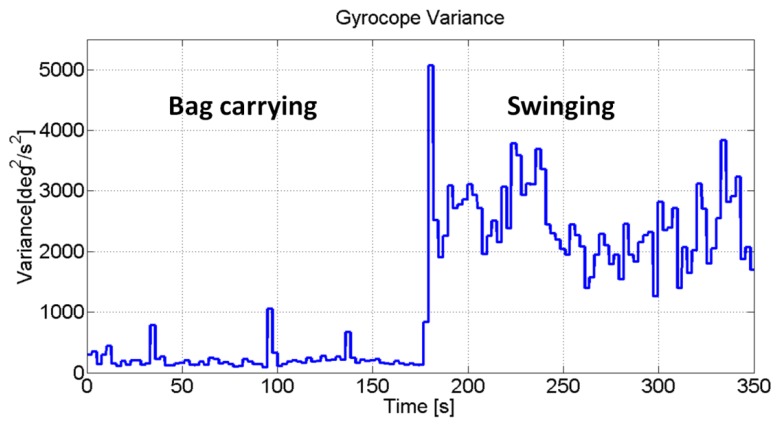
Variance of the gyroscope signal (norm) for IMU in the swinging hand and IMU in the user's bag.

In Figure 3 the variance of the gyroscope signal for a subject walking with the IMU in the bag and in the swinging hand is reported. This feature adds useful information for identifying the swinging mode, since it assumes these values, for both gyroscope and accelerometer, to be bigger than the one estimated in the texting, phoning and bag cases. Finally this feature is used for recognizing irregular motions occurring when a rapid increase of the variance is observed for inertial data without periodicity in the signals. Indeed the frequency analysis completes the motion recognition process.

#### 6.2.3. Frequency Analysis

Human walk presents a particular signature due to the periodic repetition of two main phases: the stance phase, when the foot is in contact with the ground, and the swing phase, when the foot is in the air [5]. As shown in [22], the analysis in the frequency domain of inertial signals recorded with handheld devices allows capturing the periodicity of accelerometer signals due to the user's walking activity. In fact, periodicities in the time domain produce peaks in the frequency domain. Observing the presence or absence of the above peaks, for example in the accelerometer signal, it is possible to test the signal periodicity and, subsequently, understand if the inertial force sensed by the IMU is really related to the user's walking or to a random motion of the user's hand.

**Figure 4.**
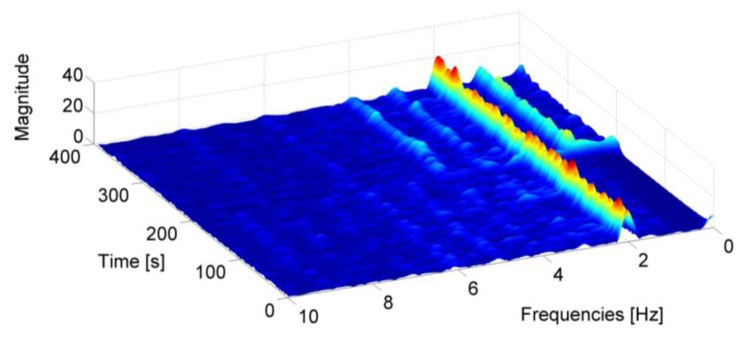
Spectrogram of the accelerometer signal for a walking user with the IMU in the hand.

The frequency analysis of the accelerometer signal is performed using the Short Time Fourier Transform (STFT) [38] in order to take into account the non-stationary nature of the signal. This technique assumes that a generic non stationary signal can be considered stationary for short periods of time. Then the spectrogram can be obtained by squaring the absolute value of the STFT. In Figure 4, the spectrogram of the accelerometer while the subject is walking with MEMS in the hand is reported. In this case the user is walking carrying the IMU alternatively in the texting mode and with the hand swinging. The periodicity of the walking is visible in the frequency peaks of the spectrogram.

**Figure 5.**
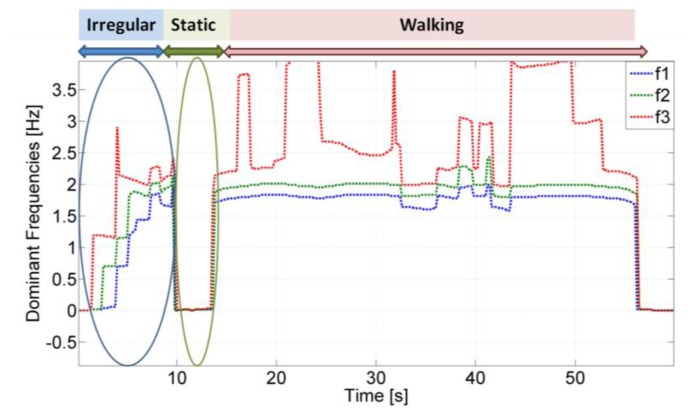
Dominant frequencies of the accelerometer signal over time. The IMU is carried in the user's hand.

In order to better analyse how these frequency peaks change over time, the three main frequencies, obtained by evaluating the first three maxima in the spectrogram, are reported over time. The estimated three dominant frequencies for a pedestrian test are reported in Figure 5 and are now further analysed.

**Figure 6.**
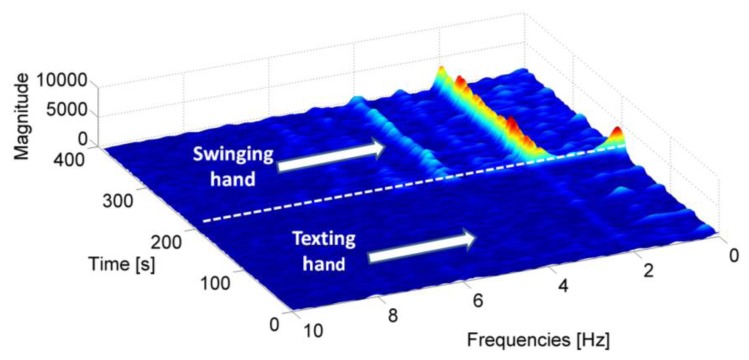
Spectrogram of the gyroscope signal for a walking user. The IMU is alternatively carried in the texting and swinging hand of the user.

When the user is walking, the first two frequencies are almost stationary since the user is not significantly changing speed. However when the user is performing an irregular motion, *i.e.*, he is looking for his mobile phone in a bag, the two main frequencies show a very irregular trend. In opposition, when the user is static the frequencies are almost equal to zero. As illustrated, the frequency analysis can be used to distinguish irregular motion modes from normal walking. Similar analysis is also performed on the gyroscope's signal while the subject is walking with a swinging hand. In fact, during hand swing a periodic rotation of the arm is observed in the gyroscope signal inducing evident peaks in the frequency domain and that would not be observed when the subject is texting or phoning. Subsequently, the presence of the above peaks can help distinguishing between different sensor carrying modes. Indeed as shown in Figure 6, for the texting cases and in opposition to the swinging mode, no frequency peak is present except in the case when the user is making a rapid change of direction.

### 6.3. Decision Making

In general a classification process can be considered as a mapping function [34] that given an input pattern characterized by a set of *d* features, **f** = [f_1_, f_2_, ⋯, f_d_] assigns each feature vector to one of *n* possible classes **c** = [c_1_, c_2_, ⋯, c_n_]. In our case, the features are the attributes defined in Section 6.1 and the classes are the user's motion modes.

Classifier algorithms are traditionally divided in two groups:

- Supervised classifier: the labelled data, whose class is known, is used to train the classifier and then to assign unlabeled data to one of the known classes.
- Unsupervised classifier: here the classes are not known a priori but are defined when the classification is completed, this is the case, for example, of the clustering classification.

For the proposed classification algorithm using handheld MEMS signals, the classes and their characteristics are defined during the classifier's design process and a supervised approach has been adopted. The classification of the user's state is performed by a decision tree classifier. The effectiveness of the decision tree classifier for motion mode recognition has been shown previously [22,33].

**Figure 7.**
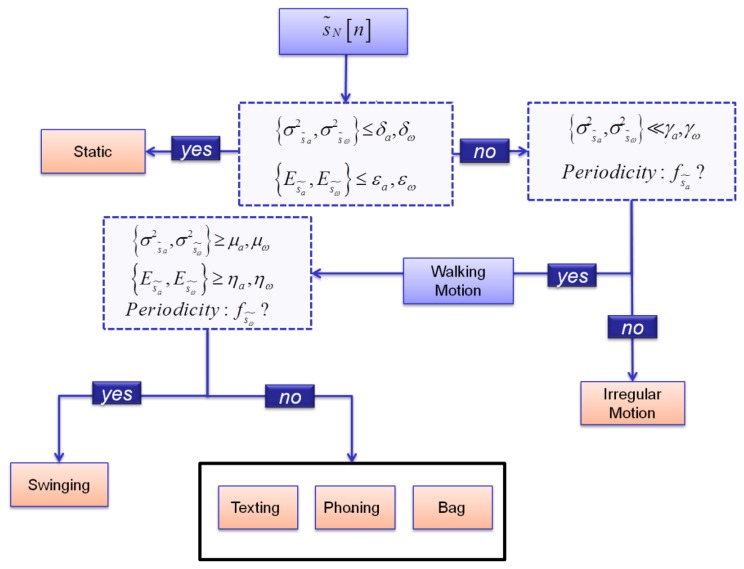
Decision tree for motion mode classification.

A decision tree is a non-parametric classifier with the form of a tree whose leaves consist of all the possible classes. In correspondence of each tree's internal node a test regarding one (univariate decision tree) or more features (multivariate decision tree) is specified. Traversing the decision tree from the root to the leaves, any input observation can be classified. The multivariate decision tree, shown in Figure 7, initially distinguishes static and dynamic activities using an approach that evaluates the energies and variances of gyroscope and accelerometer signals [33]. The periodicities of the accelerometer signal reflect the periodicities of human gait allowing recognition of the walking state. Subsequently, the gyroscope measurements are evaluated to identify walking with a swinging hand. In fact, due to the periodic rotation of the arm during the swinging mode, the gyroscope signal shows frequency peaks that are not present when the arm is almost stationary. In addition, high values of the gyroscope and accelerometer variances induced by the motion of the arm, characterize the swinging mode. Conversely, when the user is texting, phoning or walking with his/her mobile device in a bag, the arm is not moving significantly and the IMU experiences low energies and variances. The latter cases show a similar pattern for both accelerometer and gyroscope signals. Consequently, for adequately tuning the step detection algorithm proposed in Section 7, the mentioned motion modes can be considered as a unique class, as shown in Figure 7. However, it is worth mentioning that for other aims, beyond the focus of this paper, it could be of interest to assign the above motion modes to different classes. For example, different device's carrying modes, such as texting and phoning, can strongly influence the orientation information provided by the handheld sensor and, consequently, the distinction of these two states could improve the heading estimation. Finally, irregular motion modes are identified analyzing the IMU signal's variance. In fact the above class is characterized by very high values of the gyroscope and accelerometer variances in short temporal periods.

## 7. Step Detection Algorithm

Once the pedestrian motion mode is identified, it is of interest to track effective displacements by detecting steps for a pedestrian dead-reckoning navigation strategy. Indeed contrary to strapdown filtering, dead-reckoning is less sensitive to the errors inherent to low cost inertial sensors but dependant to the correct detection rate of step events. Therefore the next step toward handheld based navigation using embedded inertial sensors consists in detecting the steps performed by the user. When the sensor is placed on the user's foot, step detection can easily be performed by identifying the stance phases of the foot corresponding to zero velocity periods. This approach cannot be applied when the sensor is in the hand since in this case there isn't any period of zero velocity. However, bio-mechanic studies (e.g., [39]) show that the swinging of the arm is synchronized with the foot motion. Thanks to this relationship between arm's motion and foot's motion, from the swing of the subject's arm the step events can be detected. In fact, the periodic rotation of the arm produces an evident sinusoidal pattern in the gyroscope signal. Consequently, identifying the peaks of the gyroscope signal, the proposed algorithms are able to detect the up and forward motions of the user's hand and the synchronized swing phase of the user's foot.

During activities, such as walking on a straight line while texting a message, phoning or carrying the mobile phone in the bag, the signal recorded by the gyroscope is mainly due to the noise components and to random motions of the hand. Therefore gyroscope signals provide less useful information about the motion of the subjects and tend to perturb the detection of step events. In these cases only the accelerometer signals are used in the step detection algorithm. In fact, in these situations the accelerometer signal shows also a sinusoidal pattern due to the up and down motion of the subject's torso. In both cases, step detection can be considered as a peak detection problem. In this paper, signal peak detection is performed by recognizing a local maximum or minimum within the sliding window and using an algorithm based on adaptive thresholds. The use of adaptive threshold renders the peak detection algorithm immune to variations of the signal energy and consequently to any change of the user's pace.

**Figure 8.**
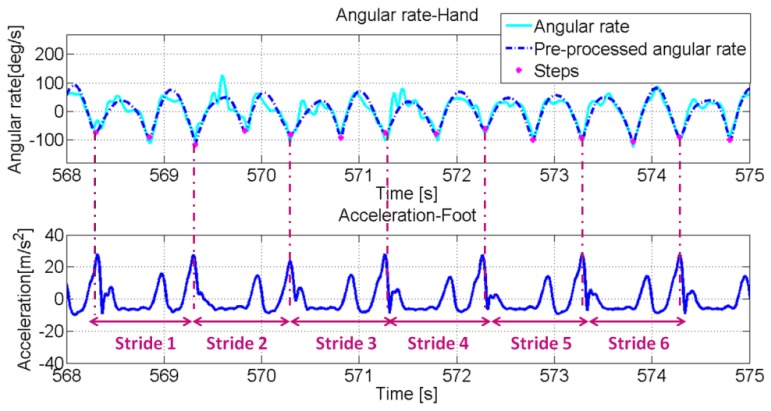
(Upper) Gyroscope signal (norm) recorded by the IMU in the swinging hand. The dots represent the detected step events. (Down)Accelerometer signal (norm) recorded by the IMU on the foot (the mean has been removed).

In addition, for minimizing the probability of false detection a dedicated pre-processing phase of IMU signals is first performed. Similar to preceding pre-filtering, accelerometer and gyroscope signals are low-pass filtered using a 10th order Butterworth filter with a 3 Hz cut-off frequency. The purpose of above pre-processing phase is to extract the signal's fundamental frequency that is induced by step events only and therefore only from an undistorted signal. Then the algorithm evaluates the maximum value within the sliding window that is assumed as a threshold value for the peak detection. In other words, if a sample in the window experiences a bigger value than the evaluated mean, a peak is identified In the upper part of Figure 8, the norm of the gyroscope signal recorded by the IMU in the swinging hand of the user is reported and the bottom part shows the norm of the acceleration signals recorded on the foot. Signals recorded in the hand and on the foot are synchronised.

**Figure 9.**
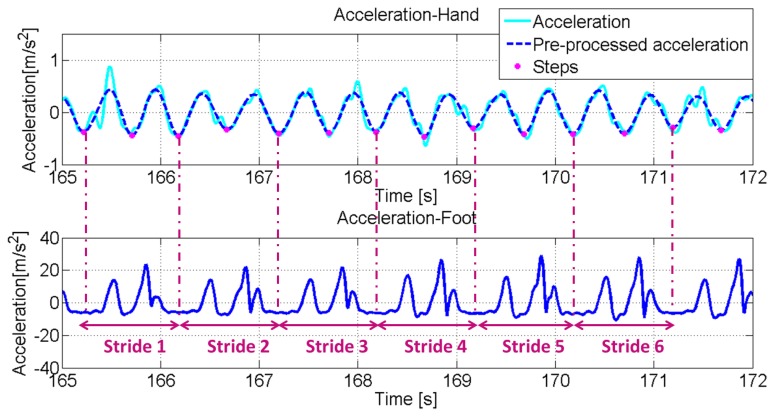
(Upper) Accelerometer signal (norm) recorded by the IMU in the texting hand. The dots represent the detected step events. (Down) Accelerometer signal (norm) recorded by the IMU on the foot (the mean has been removed).

The magenta dots mark the minima extracted from the signal. From the comparison between the signals recorded in the hand and on the foot, it is clear that these minima are directly related to the stride events that have been identified from the accelerometer signals recorded by the IMU on the user's foot. Indeed each minimum corresponds to a step event and subsequently for each couple of minimum points a stride can be identified. In Figure 9 (upper part), the norm of accelerometer signals recorded by the IMU in the texting hand is reported. Again in this case the magenta dots identify the minimum values of the signal that are related to the step events in the same way as for the swinging case.

## 8. Field Tests

**Figure 10.**
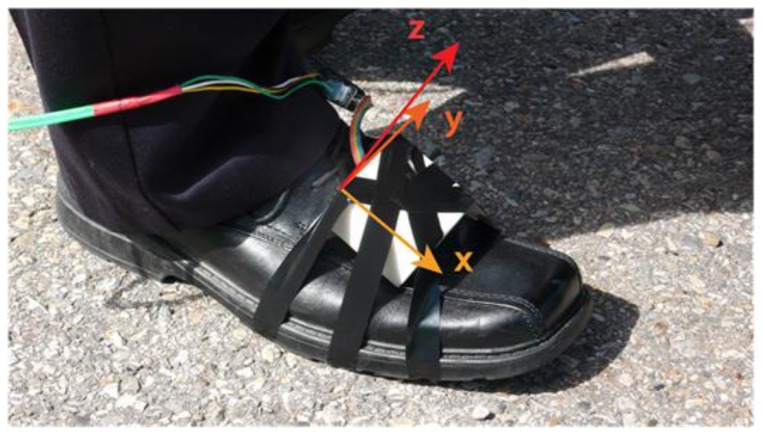
IMU attached on the foot and used as a reference for step detection assessment.

Several tests have been conducted in order to obtain a sufficient amount of data for the training and the testing phases of the designed and implemented motion mode detector. In addition, using a reference that is extracted from foot mounted IMU data, the performance of the step detection algorithm was also assessed. The data collections were performed using the Navcube, a multi-sensor navigation platform including a Novatel receiver and able to support up to 10 six-degree of freedom Analog Devices ADIS16375 inertial sensors [40]. All data are synchronised with GPS time and therefore can be used for comparative analyses. For the data collections at least two IMUs were used, one in the hand and one rigidly attached to the user's foot as shown in Figure 10.

The wires connecting the NavCube to the inertial sensors were firmly fixed to the user's body in order not to disturb the user's motion and keep natural walking gait cycles. The sensor on the user's foot was used as reference for assessing the performance of the proposed algorithms using handheld IMUs. “True” pedestrian's gait cycle was characterized using moving variances on the norm of the foot mounted accelerometer's signal [5]. Even if the identification of the stances phases of the foot by using foot mounted sensor is not immune by errors, these errors can be neglected with good approximation when the user is performing a normal walk on a flat plane. A first data collection was performed indoor for obtaining the data required for the training phase of the motion mode classifier. Two women and two men were equipped with the above hardware setup and walked along two curved routes of about 50 and 120 metres each. The subjects were requested to walk with swinging hand, then texting, phoning and carrying the IMU in a bag. Finally the subjects were requested to simulate a phone call. The users started walking with the IMU in the bag, answered a phone call while walking and then putted back the IMU in the bag. All performed motions were registered annotating the exact time of occurrence for each motion mode. This set of data was used for the training of the thresholds reported in Figure 7 in correspondence to each node of the classification decision tree. Then, two different types of field tests were performed for the algorithms assessment, namely a “free motion” data collections where the subjects could freely chose the sequence of motions to be performed and a second field test where the subjects were instructed regarding the sequence of motions. The data collections performed for the assessing stage are described in Sections 8.1 and 8.2.

### 8.1. “Free Motion” Data Collection

**Figure 11.**
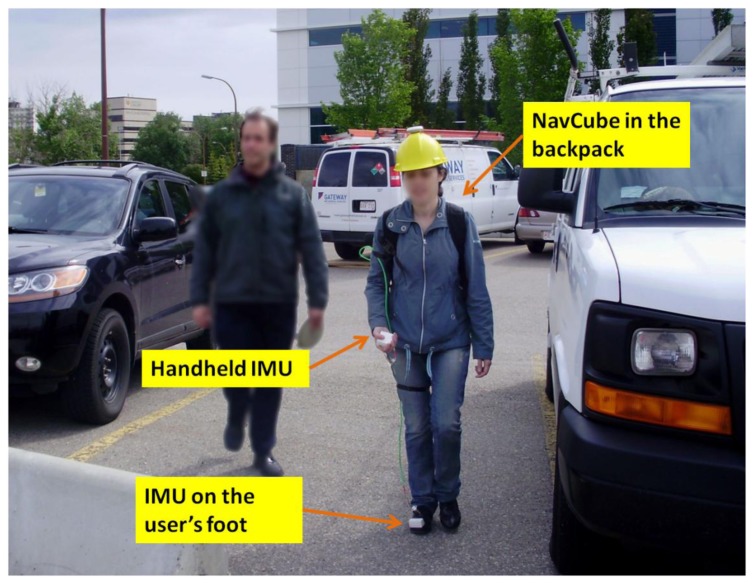

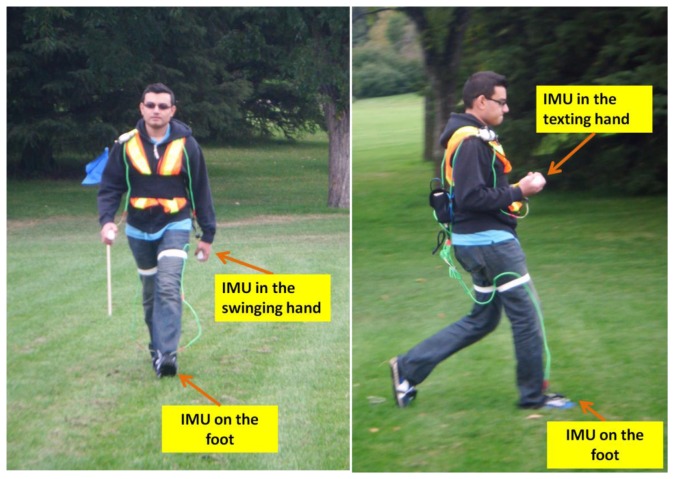
**A.** Handheld data collection setup. One IMU is located in the hand and the other one is rigidly attached to the foot. Data is recorded by the NavCube placed in the user's backpack. **B.** (**Left**) Test subject walking with the IMUs in his swinging hands; (**Right**) Test subject walking with the IMUs in his texting hands.

The “free motion” data collection was performed in a parking lot with four different subjects, two men and two women. This environment was selected to use the GPS data as reference for the user travelled path. However, it is worth underlining that since the algorithms here proposed are only based on accelerometer/gyroscope data the same performances would be obtained indoor. As shown in Figure 11, the test subjects, equipped with the same hardware used for the training phase, were requested to perform a free motion. Practically, pedestrians were walking over a distance of about 300 metres freely carrying the IMU in their hand without following instructions about the sensor's orientation and freely choosing one among the carrying modes described in Section 5. This “free motion” data collection was performed with the purpose to test a realistic situation. The subjects were nevertheless requested to perform all considered motion modes. Again the time of occurrence of each motion mode was carefully annotated. This last data set was used to test the performance of the motion mode classifier reported in Section 6.

#### 8.1.1. Motion Mode Classifier Performance

The performances of the motion mode detector are summarized in the confusion matrix given in Table 1. The rows of the confusion matrix report the performed activities while the columns report the predicted activities. Along the principal diagonal of the confusion matrix, the accuracy (in light blue), *i.e.*, that is the percentage of correctly classified samples, is reported for each motion mode. Instead along the off diagonal elements the percentages of misclassified activities are reported (in green). The percentage of occurrence for each motion mode is also reported.

Globally the proposed motion mode recognition algorithm based on signals collected with handheld IMU classifies the activity in the correct category more than 94% of the time no matter what activity is performed by the pedestrian.

The percentage of irregular motions detection is equal to 94%. As previously explained, when an irregular motion is identified the step detection algorithm is not applied, since this state does not correspond to a real change of geographical user position. Consequently, the identification of this class is essential to avoid wrongly propagating the user's position leading to unbounded position errors in any PDR algorithm. However, from Table 1, one can observe that 6% of irregular motions are wrongly classified as swinging motion. The explanation comes from the fact that swinging and irregular motions have the closest features, therefore when irregular motion happens during hand swing, it may be miss detected. The analysis of the periodicities of the gyroscope signal for both motion modes allows reducing this ambiguity in most of the cases. Indeed one motion mode is periodic whereas the other is expected to be random. In these 6%, the step detection will still be applied in case where it should not. The consequence is that false steps might be detected, leading to non-existing displacement. However the step detection algorithm described in Section 7 relies on adaptive thresholds that will mitigate this impact. The error induced in the PDR navigation solution will be proportional to the amount of false detected steps.

Moreover, 3% of swinging cases and 3% of texting/phoning/bag carrying cases are wrongly identified as irregular motion. Such miss classifications will also affect the propagation of the user position, since in these cases the step detection algorithm will not be applied, keeping the navigation solution unchanged instead of potentially moving few steps. Again the error will be proportional to the amount of miss-detected steps.

Globally the test confirms that the identification of an irregular motion state with a handheld device is the most challenging task but also a critical point for achieving good performance in inertial PDR navigation. Indeed, the kinds of irregular motions performed by the user are unpredictable and various, rendering their identification a difficult task. Further investigations could be performed to reduce this misclassification percentage by increasing the types of irregular motions analyzed in the training phase. For the texting/phoning/bag modes, it has been observed that the majority of misclassifications occurred when the pedestrian was changing direction.

When the swinging case is wrongly identified as a texting, phoning or bag carrying case, the step detection algorithm is still applied but by exploiting the accelerometer signal instead of the gyroscope signal. Although it has been empirically found that gyroscope measurements constitute a more robust information source for detecting steps in the swinging case, the accelerometer signal still allows the identification of step events. The accuracy gets slightly reduced especially in the presence of irregular motions. Conversely, the probability of error in the step detection algorithms and consequently in the final position computation could increase if the swing state is identified instead of the texting, phoning, or back carrying state. In these cases, the step detection algorithm could be applied to the angular rates providing potential noisy information unrelated to the user gait events.

Overall the observed maximum percentage of motion state misclassification is 6%. For a 10-minute walk, this corresponds to 36 miss or false detected gait cycles with a two-step per second frequency. The maximum induced localization error is 7.2% of the travelled distance with a 1.2 m/s average walking speed. However this number is overstated since the step detection algorithm is still able to successfully detect steps when misclassification of motion state occurs, as previously discussed.

Finally, it should be considered that carrying the NavCube in a backpack and the sensor mounted on the user's foot could affect the characteristics of the analysed motion modes and, subsequently, condition the motion mode recognition process. For this purpose, a specific test has been conducted using only a handheld inertial sensor. This analysis has allowed observing that the only motion mode affected by the test equipment is walking with the sensor in the swinging hand. Specifically, in some cases the amplitude of the arm's swing is decreased when the user carries the backpack with the test equipment. However, also in these cases the gyroscope's variance is significantly bigger than the one induced by other motion modes and, subsequently, the performance of the motion mode classifier is not affected.

#### 8.1.2. Step Detection Algorithm Performance

The data collected have been also used to assess the proposed algorithms for detecting steps using inertial signals collected with handheld MEMS. Several statistics have been extracted by comparing the step events detected from handheld device with the ones from the foot mounted IMU. All results are summarized in Table 2.

The probability of detection (P_det_), the probability of false alarm (P_fa_) and the probability of miss detection (P_md_) have been evaluated. The probability of detection reflects the percentage of correctly identified samples as compared with the reference from the foot. The probability of false alarm gives the percentage of incorrectly identified samples. The probability of miss detection is given by the percentage of samples that are missed. The percentage of occurrence of each motion mode is also reported. The results for each motion mode are summarized in Table 2 where we can see that for each motion mode the performance of the algorithm is higher than 98%.

It is important to highlight that the adaptivity of the algorithms to the identified motion mode renders the performances of step detection process nearly immune to the nature of the action performed by the pedestrian both in terms of global locomotion and hand's motion.

### 8.2. “Controlled” Data Collection

To further confirm the validity of the algorithms another extensive data collection has been performed. This time the subjects were required to perform the activities in a specific sequence. In order to increase the robustness of the classification assessment, the subjects used for this data collection were different from the ones who performed the free data collection described in Section 8.1. Specifically, five women and five women carrying again the Navcube at the waist, with one sensor on the foot and one in the hand, walked twice along a route of around 300 m, for a total path length of about 600 m. The same number of male and female subjects was chosen for testing the effectiveness of the algorithms regardless of gender. As shown in Figure 11, in the first route, the user had to walk carrying the sensor in swinging mode while during the second route the subjects were asked to keep the sensor in texting mode. The sequence of motions to be performed has been a priori defined in order to allow a consistent comparison of the results obtained for all subjects. However, in order to maintain the scenario realistic each test subject could perform the considered motion in the preferred way. Indeed, again any indication was given about the sensor orientation and, for example, the subjects could choose if perform the texting mode using one or both hands.

Furthermore, the texting mode is representative also of the phoning and bag carrying states, since all these modes have been included in unique class, as detailed in Section 6. The subjects were performing the two modes without stopping in order to do not affect their natural walking style. Finally, the same number of female and male subjects was selected in order to guarantee the effectiveness of the algorithm regardless of walking characteristics related to the gender of the subject. The performance of the motion mode classifier for this data collections have been reported in Table 3. Again the high probabilities of correct motion identification are reported along the principal diagonal (in light blue). Also for this test the probabilities of misclassification, shown by the off diagonal elements (in green), are very low. Moreover, in Table 4 the performance of the motion mode detector and the step identification algorithms are reported for each test subject. The male subjects are indicated with the letter M and the women with the W. The algorithms achieve high performance regardless of the sensor position for all 10 subjects.

## 9. Conclusions

Algorithms for characterizing the gait of pedestrian using accelerometer and gyroscope signals recorded in a handheld device have been developed and presented. They consist of two parts. First the motion mode, both in terms of global locomotion but also hand motion, is identified. Second step events are detected. For improving the robustness of step detection in a context where the hand is undergoing a lot of motion, a priori knowledge about the user's motion state is used. For this purpose, before detecting step events, a decision tree classifier able to recognize several motions, including phoning texting and walking with swinging hand is applied. Moreover, an important state corresponding to irregular motion that is often ignored in motion classification algorithms using handheld IMUs has been investigated. This state is of particular interest as it does not contribute to the pedestrian displacement. For the purpose of motion classification, several features in the frequency and time domains have been extracted from the tri-axis accelerometer and tri-axis gyroscope signals. Novel step detection algorithms using handheld device and their capacity of adapting to the detected motion mode have been presented. Experimental tests using MEMS sensors, conducted with four test subjects for the training phase and four other test subjects for the final assessment show a percentage of correct classification above 95% and of correct step event detection above 97% for all motion modes here considered. The step events extracted using the proposed algorithm together with a step length model allow propagating the user position by applying a PDR algorithm for handheld devices [13]. These results open new research options toward free-inertial tracking of pedestrian using handheld inertial sensors, which are already widely embedded in smartphones nowadays.

## Figures and Tables

**Table 1.** Confusion matrix for the motion mode classifier in the **“**free motion” data collection.

**Activity**	**Classified as:**		

	**Swinging**	**Texting/Phoning/Bag**	**Irregular motion**
**Occurrence**	42%	53%	5%
**Swinging**	**95%**	2%	3%
**Texting/Phoning/Bag**	1%	**98%**	3%
**Irregular motion**	6%	0%	**94%**

**Table 2.** Step detection algorithm performance for the “free motion data collection”.

**Steps in…**	**…hand swinging**	**…texting**	**…phoning**	**…bag**
	
**Occurrence**	**42%**	**25%**	**20%**	**8%**
**P_det_**	**99%**	**100%**	**99%**	**97%**
**P_fa_**	**1%**	**0%**	**0%**	**1%**
**P_md_**	**1%**	**0%**	**1%**	**3%**

**Table 3.** Confusion matrix for the “controlled” data collection [13].

**Test Motions**	**Classified as:**		

	**Texting**	**Swinging**	**Irregular**
**Texting**	**100%**	0%	0%
**Swinging**	0%	**98%**	2%

**Table 4.** Step detection algorithm performance for the “controlled” data collection [13].

**Subject**	**%P_det_(motion)**	**%P_det_(steps)**

**M1**	100	99
**M2**	100	100
**M3**	99	100
**M4**	100	99
**M5**	100	100
**W1**	98	97
**W2**	100	100
**W3**	98	99
**W4**	100	98
**W5**	100	100
**Mean**	**99.6**	**99.2**
